# Perforation of gastroesophageal junction, stomach, and diaphragm following blunt abdominal trauma: A near miss: A case report

**DOI:** 10.1016/j.ijscr.2021.105786

**Published:** 2021-03-17

**Authors:** Dinesh Prasad Koirala, Bibek Man Shrestha, Ankush Kansal, Diptee Poudel, Subita Neupane, Geha Raj Dahal

**Affiliations:** a Pediatric Surgery Unit, Tribhuvan University Teaching Hospital, Institute of Medicine, Kathmandu, Nepal; b Maharajgunj Medical Campus, Institute of Medicine, Kathmandu, Nepal; c Department of GI and General Surgery, Tribhuvan University Teaching Hospital, Institute of Kathmandu, Nepal; d Department of General Medicine and Emergency, National Academy of Medical Sciences, Bir Hospital, Kathmandu, Nepal

**Keywords:** Blunt abdominal trauma, Gastro-esophageal junction, Perforation

## Abstract

**Introduction:**

Blunt abdominal trauma causing Gastro-esophageal junction (GEJ), diaphragm, and gastric perforation in children is a very rare occurrence. However, the injury is serious and life-threatening with significant morbidity and mortality.

**Presentation of case:**

We report an unusual case of a 14-year-old boy with an accidental fall from the tree with blunt abdomen trauma with a perforating injury to GEJ, stomach, and diaphragm.

**Discussion:**

Multiple vital organs in the abdominal cavity are vulnerable to damage in blunt abdominal trauma. The rarity of perforation, diagnostic delay, and early septic occurrence sums up to higher patient morbidity and mortality. A high degree of suspicion and urgent laparotomy and surgical repair forms the cornerstone in management.

**Conclusion:**

Early suspicion along with effective resuscitation and early laparotomy and surgical repair is crucial for survival and optimal outcome of the patient.

## Highlights

• Perforating intra-abdominal injuries are often fatal.
• We report perforating intra-abdominal organ injuries with rare GEJ perforation despite blunt trauma to the abdomen.
• Early suspicion and laparotomy to manage all the potential injuries along with effective resuscitation and prehospital care are critical.
• Esophageal perforations are common with high velocity automobile accidents, crush injuries and endoscopic interventions.
• Most common perforations occur along the cervical esophagus followed by distal esophagus.

## 1 Introduction

High-velocity automobile accidents crush injuries and endoscopic interventions share a major etiological proportion for traumatic esophageal perforation while other modes are uncommon precedence. Within the esophageal tube, most of the perforations occur along the cervical esophagus, followed by the distal esophagus [[1], [2], [3], [4]]. Indeed, Gastro-esophageal junction (GEJ) perforation combined with gastric perforation is a rare occurrence following blunt trauma presumably because of their low mobility within the abdominal cavity. We report a case of a 14-year-old child who had perforating injuries to GEJ, esophagus, and stomach despite blunt abdominal injuries to the abdomen and chest. We believe the details of our case merits attention because of its unique nature regarding the nature of injury and outcome. The case has been reported in line with the SCARE criteria [5].

## 2 Presentation of case

**Fig. 1:**
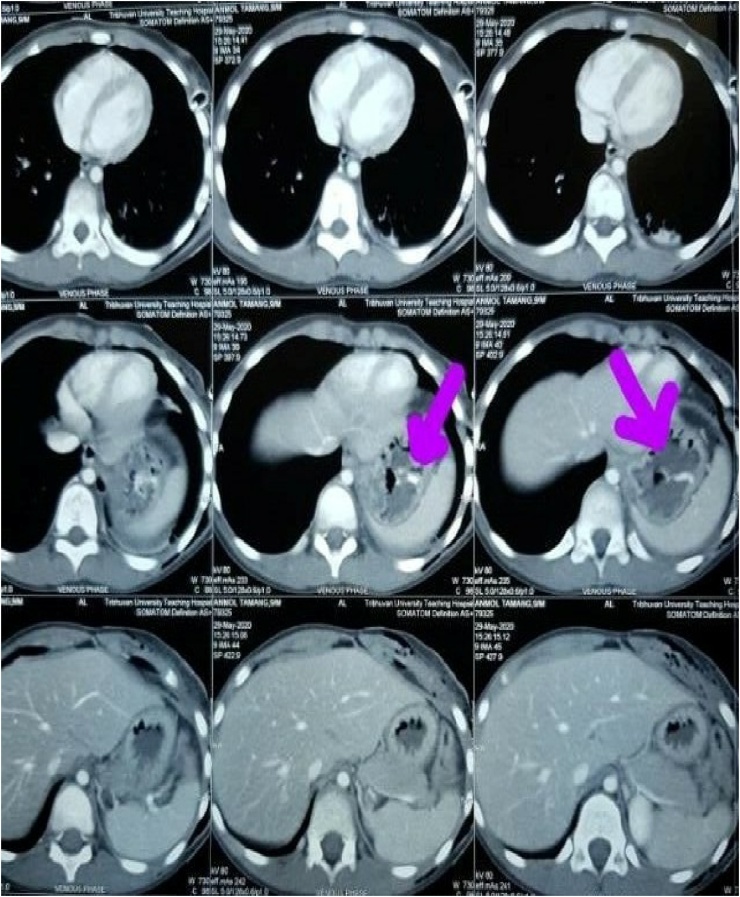
Preoperative CT scan show perforations.

**Fig. 2:**
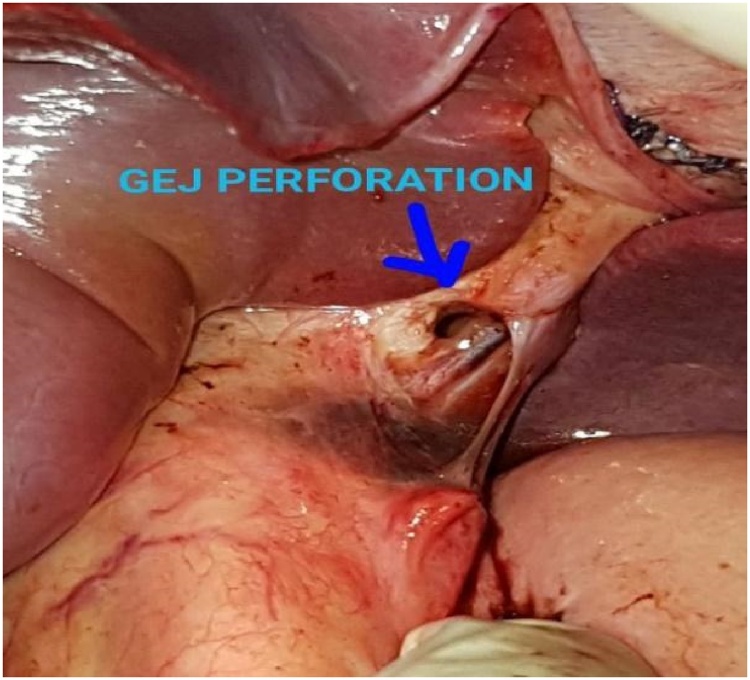
Gastroesophageal Perforations (0.5cm × 0.5 cm).

**Fig. 3:**
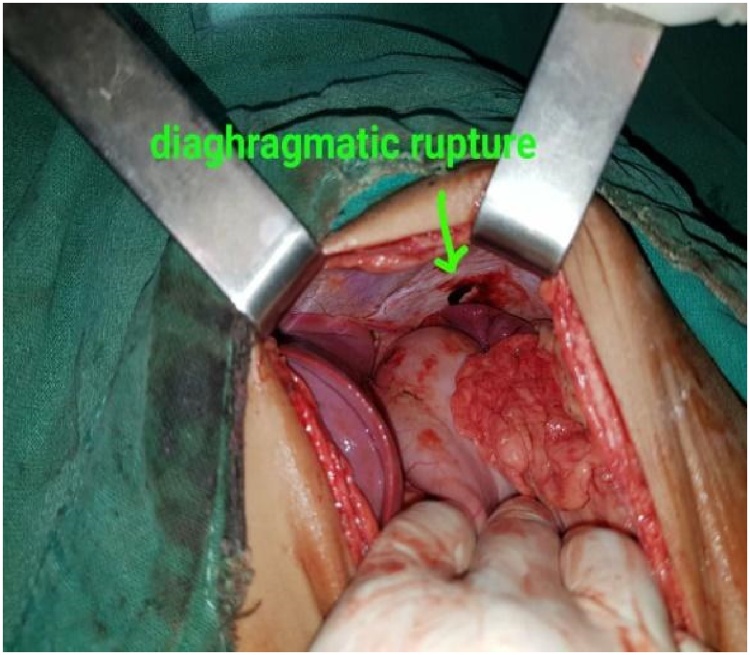
Diaphragmatic rupture measuring 2cm × 3 cm.

**Fig. 4:**
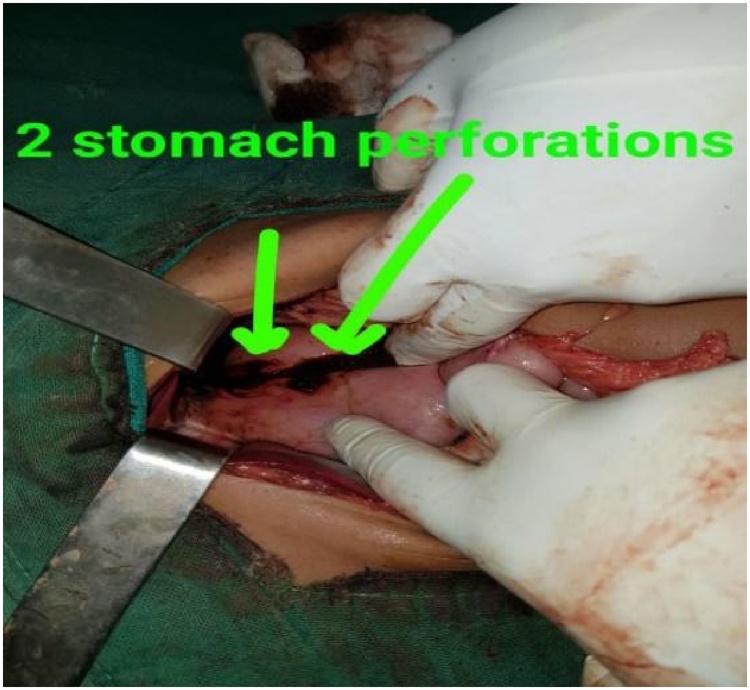
Perforations at stomach each measuring 1cm × 1cm.

**Fig. 5:**
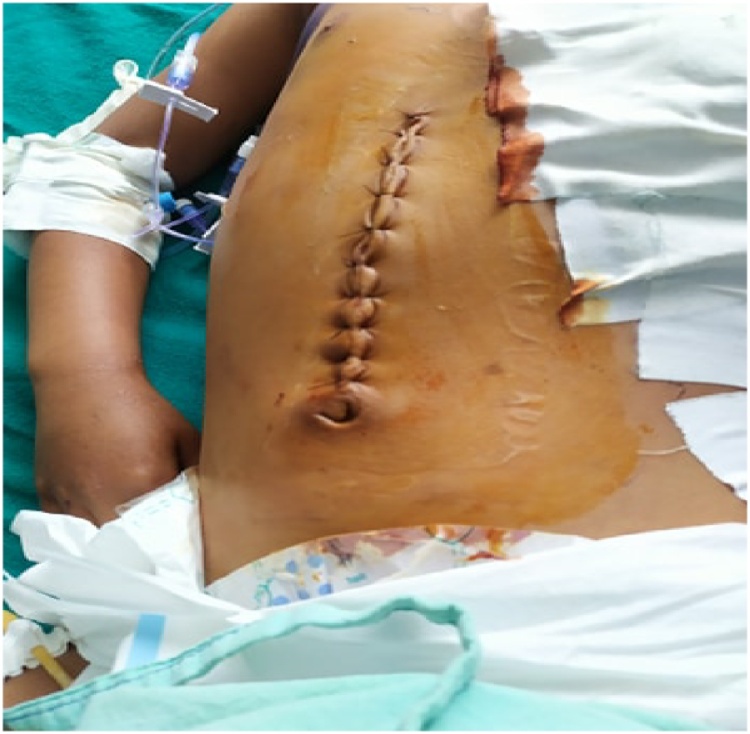
Upper Midline incision and repair.

A previously healthy 14-year-old boy, with no significant drug and family history, was brought to the emergency department shortly after an accidental fall from a tree. He had fallen prone over uneven earth with anterior chest and abdomen noted as the primary site of impact. On arrival to our Emergency Department, the patient was conscious, tachycardic (HR-140bpm), blood pressure of 120/80 mmHg, and other vitals stable with no sign of respiratory distress. The patient did not have any relevant past history. On local examination, penetrating chest injury measuring 4 cm was noted along the left anterior chest locating 3 cm below the left nipple. A thorough evaluation revealed left pneumothorax and a 28 F chest tube was inserted into the left pleural space and the tube kept in underwater drainage. The child was kept in close observation with fluid resuscitation, empirical broad-spectrum antibiotics, and optimal pain management. During the second day of his hospital stay, he vomited out reddish blood and passed black tarry stool on multiple occasions. Suspecting traumatic gut injury an esophagogram and CECT abdomen and pelvis were done which revealed focal collection and dye extravasation respectively (Fig. 1). The child was taken to the OR and an exploratory laparotomy was done via an upper midline approach (Fig. 5). On the operating table, perforations were visualized over GEJ, stomach, and duodenum (Fig. 2, Fig. 3, Fig. 4).

The perforations were repaired and the surgical wound was closed with a drain kept in-situ. The child was observed in an intensive care unit with restriction of oral intake, fluid resuscitation, and serial follow-up of hemoglobin, packed cell volume, broad-spectrum antibiotics and adequate pain management without any ionotropic support. He was discharged after 10 days of hospital stay with an uneventful postoperative period. In the follow-up of 3 months, there was no issue regarding feeding. The procedure was performed by the experienced pediatric surgeons’ team of Tribhuvan University Teaching Hospital encountering no other complications and was well tolerable by the patient and was happy with the procedure.

## 3 Discussion

Most of the esophagogastric perforations coming to medical attention are due to iatrogenic causes following GI instrumentation [4]. A history of a high-velocity automobile accident and crushing injury is usually present in patients who suffer non-iatrogenic esophageal and gastric perforations, though it might be caused by seemingly minor insults like an inappropriate Heimlich maneuver [6]. Distal esophageal perforations an exceedingly rare occurrence in blunt abdominal trauma owing to its relatively protected habitat in the abdominal cavity uninfluenced by the acceleration-deceleration forces. The cervical esophagus is the one to give out the most due to its relatively higher mobility within the fascial compartments of the neck [7]. Gastric perforations follow similar modes of accidents; the victims usually would have suffered poly-trauma; hence these perforations are missed behind the curtain set by more serious injuries in the absence of obvious signs pointing to them. Even if early laparotomy is attempted the gastric perforations tend to get missed owing to the subtlety of wounds [8]. They come to the attention of the physician only after septic sequelae sets in. The septic collection along the cervical esophagus is relatively contained within the closed fascial compartments of the neck hence the patient morbidity and mortality are relatively low in contrast to the distal esophagus and stomach where mediastinitis and peritonitis sets in early and rampantly. The rarity of perforation, diagnostic delay, and early septic occurrence sums up to higher patient morbidity and mortality.

There are two proposed mechanisms of perforation following such injuries. The rise in intraluminal pressure against closed glottis creates a blast effect whereas the acceleration-deceleration forces acting on the victim led to the disruption of the feeding vessel followed by ischemia and necrosis of the gut wall. In the absence of other symptoms confounding the diagnosis patients usually complain of chest or abdominal pain, dyspnea, or dysphagia depending upon the site of rupture [4]. Our patient had not reported any of the aforementioned symptoms; suspicion of esophageal perforation was made due to the passage of blood-mixed vomitus and melena. Contrast esophagogram, UGI endoscopy, and CECT abdomen are the investigative options. Contrast esophagograms show leaks from the ruptured segment though the false-negative rate is as high as 10%. Gastrografin as contrast material is used first, if results come negative then barium can be used. If the treating physician suspects perforation despite the absence of contrast leakage, urgent laparotomy, and exploration is mandated owing to its high false-negative results [9].

The various treatment options include primary surgical closure, operative drainage, non-operative drainage, and resection. If the diagnosis is made within the first 24 h then primary surgical closure is the best option; if diagnosed late patient morbidity and mortality is significantly increased independent of treatment options [[10], [11], [12]]. Moreover, patients might suffer additional complications requiring surgical interventions following delayed suture repair. However, the majority of iatrogenic perforation of the esophagus can be managed conservatively with pleural drainage [13]. Therefore a high degree of suspicion is required for early diagnosis and prompt management of esophagogastric perforation to significantly improve patient morbidity and prevent mortality.

## 4 Conclusion

Though high-velocity automobile accidents, seat belt injuries, and crush injuries are cited as the major non-iatrogenic causes of esophageal-gastric perforations, it should still be suspected in other non-peculiar blunt traumatic situations. An esophagogram will reveal leakage from the ruptured segment. If perforations are suspected despite negative contrast studies an early exploration and primary surgical repair within 24 h of injury is recommended to improve patient survival and avoid complications related to delayed treatment.

## Declaration of Competing Interest

The authors report no declarations of interest.

## Sources of funding

None.

## Ethical approval

Nothing to declare.

## Consent

Written informed consent was obtained from the patients’ father for publication of this case report and accompanying images. A copy of the written consent is available for review by the Editor-in-Chief of this journal on request.

## Author contribution

Dinesh Prasad Koirala (DPK), Ankush Kansal (AK) and Gehraj Dahal (GD) = Study concept, Data collection, and surgical therapy for the patient.

DPK, AK and Bibek Man Shrestha (BMS) = Writing - original draft preparation.

BMS and Diptee Poudel (DP) = Editing and writing.

DPK and GD = Senior author and manuscript reviewer.

All the authors read and approved the final manuscript.

## Registration of research studies

Not applicable.

## Guarantor

Dinesh Prasad Koirala.

## Provenance and peer review

Not commissioned, externally peer-reviewed.
